# Prediction of Bacterial Etiology in Pediatric Patients with Acute Epididymitis: A Comparison of C-Reactive Protein and Urinalysis in Terms of Diagnostic Accuracy †

**DOI:** 10.3390/biomedicines12122866

**Published:** 2024-12-17

**Authors:** Kang Liu, Chi-Shin Tseng, Shin-Mei Wong, Kuo-How Huang, I-Ni Chiang, Chao-Yuan Huang, Chih-Hung Chiang

**Affiliations:** 1 Department of Education, National Taiwan University Hospital, National Taiwan University, Taipei 100229, Taiwan; 2 Department of Urology, National Taiwan University Hospital, National Taiwan University, Taipei 100229, Taiwan; 3 Division of Urology, Department of Surgery, Taoyuan General Hospital, Ministry of Health and Welfare, Taoyuan 33004, Taiwan; 4 Department of Research and Development, Taoyuan General Hospital, Ministry of Health and Welfare, Taoyuan 33004, Taiwan

**Keywords:** acute epididymitis, pediatric, bacterial infection, C-reactive protein, urinalysis, antibiotics

## Abstract

**Background/Objectives**: We aimed to determine the proportion of bacterial etiology in pediatric acute epididymitis (AE) and to compare the predictive accuracy of C-reactive protein (CRP) and urinalysis. **Methods**: Pediatric patients diagnosed with AE in National Taiwan University Hospital from 2009 to 2018 were retrospectively identified. Patient profiles, including clinical symptoms, physical findings, laboratory data, and treatment types, were collected. Patients were categorized into acute bacterial epididymitis (ABE) or acute non-bacterial epididymitis (ANBE) groups based on the presence or absence of bacterial growth in urine cultures. The primary endpoints were the proportion of patients with ABE and those who received antibiotic therapy. The secondary endpoint was to assess the diagnostic accuracy of CRP and urinalysis for ABE. **Results**: The final cohort comprised of 289 patients, of whom 216 (74.7%) received antibiotics. Urine culture was obtained for 167 (57.8%) patients, and 52 (31.1%) were positive for a bacterial source. The median CRP and positive rate for urinalysis were significantly higher in the ABE group compared to the ANBE group (CRP: 3.68 vs. 0.25 mg/dL; *p* < 0.001; urinalysis: 41% vs. 23%; *p* = 0.005). Multivariate analysis revealed that elevated CRP was significantly associated with AE (odds ratio [OR], 61.96; *p* < 0.001), whereas positive urinalysis was not (OR, 2.09; *p* = 0.33). The area under the receiver operating characteristic curves for CRP was higher than that for urinalysis (0.82 vs. 0.72). **Conclusions**: Serum CRP proved to be a more accurate and reliable tool than urinalysis for predicting pediatric ABE. This could provide guidance to practitioners when prescribing antibiotics in the future.

## 1. Introduction

Pediatric acute epididymitis (AE) commonly manifests with a rapid onset of scrotal pain and swelling, warranting differentiation from other emergent conditions such as spermatic cord torsion, testicular appendage torsion, and orchitis. The possible causes of AE include bacterial or viral ascension via the urinary tract, post-infectious inflammation, and trauma [1]. With the advancement and widespread adoption of ultrasonography in diagnosing acute scrotal conditions, recent published series have revealed a higher incidence of pediatric AE than previously reported [2,3,4,5]. According to current European Association of Urology guideline on AE [6], urine culture should be performed and investigation for lower urinary tract abnormalities may be required. For non-sexually active men, a single antimicrobial agent (fluoroquinolone) is recommended.

However, in contemporary clinical practice, challenges emerge in both the performance and interpretation of scrotal ultrasonography in children [7], and urine cultures are not routinely conducted. Despite multiple studies demonstrating that the positive rates for urinalyses and urine cultures are extremely low in pediatric AE [8,9,10,11], prophylactic antibiotics remain the standard treatment for most of the patients. Therefore, in recent years, the necessity of antibiotics in the management of pediatric AE has been an ongoing debate. We aimed to evaluate the predictive factors for acute bacterial epididymitis (ABE) and assess the accuracy of urinalysis and C-reactive protein (CRP) as initial measures for differentiating ABE from acute non-bacterial epididymitis (ANBE).

## 2. Materials and Methods

### 2.1. Study Population and Clinical Data

Patients with a diagnosis of epididymitis in the electronic medical records at National Taiwan University Hospital (NTUH) between 1 January 2009 and 31 July 2018 were retrospectively reviewed. All patients were evaluated by board-certified urologists. AE was diagnosed clinically according to findings involving the scrotum, including redness, swelling, and tenderness. Doppler ultrasonography showing an enlarged epididymis and increased blood flow was also used to support the diagnosis, but it was not mandatory for diagnostic criteria. Patients with other etiologies of acute scrotum, including recent urologic intervention, testicular torsion, or torsion of the appendix testis, and those aged ≥18 years, were excluded. A total of 289 pediatric AE patients were included in the final cohort. The institutional review boards of NTUH granted approval for this study (IRB #201704024RINA).

We collected patient data, including symptoms, physical findings, underlying conditions, disease laterality, laboratory data, and treatment course. Laboratory studies included urinalysis, urine culture, serum white blood cell (WBC) count, and serum CRP. An abnormal urinalysis was defined as ≥5 WBC/high-power field (HPF). A positive urine culture was defined as ≥100,000 colony-forming units (CFU)/mL in midstream urine and 10,000 CFU/mL in clean catheterization urine. Serum high-sensitivity C-reactive protein (hsCRP) levels were measured using the turbidimetric immunoassay method, which was initiated based on the clinical assessment of the physicians at the emergency department when pediatric patients presented with suspected infections. Patients were sorted into ABE or ANBE groups according to positive or negative pathogen growth from urine culture. The primary endpoint was the prevalence of ABE and the percentage of patients who had received antibiotic therapy. The secondary endpoint was to evaluate the predictive factors for ABE.

### 2.2. Statistical Analysis

Continuous variables, summarized as medians with interquartile ranges (IQRs) or means with standard deviations (SDs) according to their distribution, were analyzed using the Wilcoxon rank sum test or Student’s *t*-test, respectively. Categorical data, presented as counts and percentages, was compared using the χ^2^ test.

For determining the risk factors of ABE, odds ratios (ORs) with 95% confidence intervals (CI) were calculated. Statistically significant or clinically important factors in the univariable analysis were included in a multivariable model for further analysis. The predictive capability of the variables was assessed by calculating the area under the receiver operating characteristic (ROC) curves. All statistical analyses were performed using the SPSS statistical software (version 22.0; IBM Corp, SPSS, Inc., Chicago, IL, USA). Statistical significance was defined as a two-tailed *p* < 0.05.

## 3. Results

A flow diagram for the 289 patients in this cohort is shown in Figure S1. Baseline characteristics of the final cohort are summarized in Table 1. In this cohort, the mean age was 9.5 ± 5.2 years, with 34 (11.8%) patients presenting with recurrent episodes. Positive urinalyses were discovered in 53 (25.2%) patients. Of the 167 patients tested with urine cultures, 52 (31.1%) were positive for a bacterial source. In total, antibiotic therapy was administered to 216 (74.7%) patients. In addition, unilateral epididymitis occurred in most of the cases, with equal involvement of both sides. Patients with concomitant urological diseases included balanoposthitis, phimosis, hypospadias, urethral diverticulum, posterior urethral valve, retractile testis, undescended testis, hydrocele, and testicular tumor. Upon presentation to the hospital, the most common symptoms in descending order of prevalence were epididymal tenderness, scrotal swelling, and scrotal erythema, with 17 (14.5%) patients having fever up to 38 °C. Furthermore, mild elevations of serum WBC count (median [IQR], 10.56 [8.09–14.65] 10^3^/μL) and CRP (median [IQR], 0.8 [0.09–3.54] mg/dL) were revealed.

Compared to the ANBE patients, ABE patients were significantly younger (mean ± SD, 6.8 ± 5.2 vs. 9.3 ± 5.5; *p* = 0.02) and had a higher prevalence of tenderness of epididymis (82.7% vs. 84.3%; *p* = 0.02). A higher rate of positive urinalysis (41.2% vs. 23.1%; *p* = 0.005) and elevated CRP level (median [IQR], 3.68 [1.56–8.45] vs. 0.25 [0.05–1.49] mg/dL; *p* < 0.001) were observed in the ABE group compared to the ANBE group.

**Figure 1:**
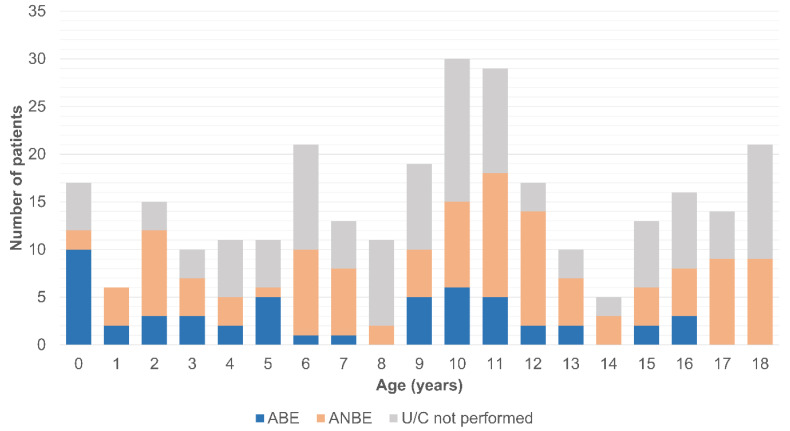
Age distribution of patients with acute epididymitis. ABE, acute bacterial epididymitis; ANBE, acute non-bacterial epididymitis; U/C, urine culture.

The distribution of the ages at which patients were diagnosed with AE is illustrated in Figure 1. The distribution demonstrated a bimodal pattern, with peaks in infancy and the prepubertal period, followed by a progressive increase after 15 years of age.

Table 2 presents the results of univariate and multivariate logistic regression analyses. While age (OR, 0.89; 95% CI, 0.83–0.94; *p* = 0.001) and positive urinalysis (OR, 2.32; 95% CI, 1.14–4.75; *p* = 0.02) were associated with ABE in the univariable analysis, elevated serum CRP was the only factor (OR, 61.96, 95% CI, 6.10–629.01; *p* < 0.001) that was independently associated with ABE.

**Figure 2:**
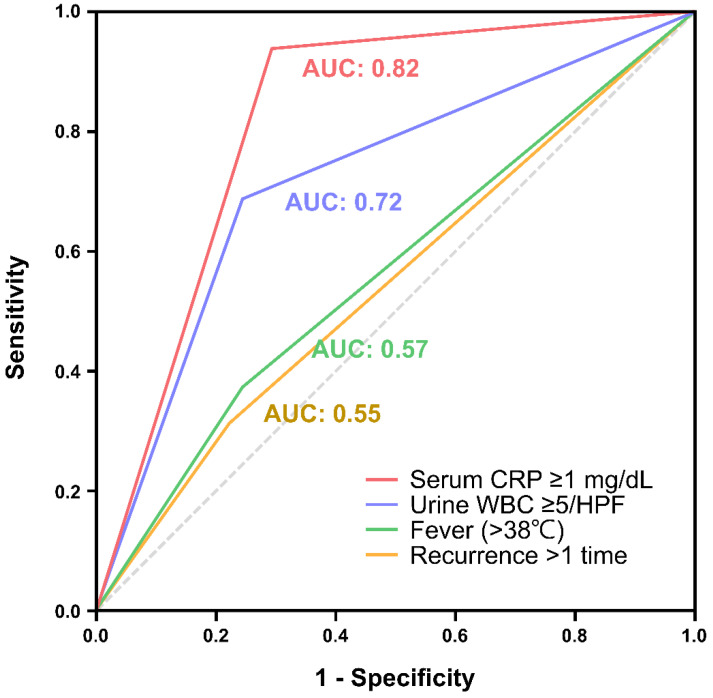
Receiver operating characteristic (ROC) curves for predictors of ABE. ABE, acute bacterial epididymitis; AUC, area under the curve; CRP, C-creative protein; HPF, high-power field; WBC, white blood cells.

The diagnostic accuracy of the factors associated with ABE was estimated by ROC curves (Figure 2). The area under the curve (AUC) for elevated serum CRP reached 0.82, followed by 0.72 for positive urinalysis, and 0.57 and 0.55 for fever and recurrence, respectively. Table 3 demonstrates the strengths and limitations of urinalysis and CRP in diagnosing pediatric ABE patients.

The pathogen spectrum in urine cultures was predominantly composed of Gram-negative bacilli (59.6%), with *Escherichia coli* accounting for the highest proportion (Table S1). Other pathogens identified included *Pseudomonas aeruginosa*, *Klebsiella* spp., *Enterococcus* spp., *Streptococcus viridans*, *Staphylococcus* spp., *Citrobacter koseri*, *Enterobacter cloacae*, *Morganella morganii*, *Serratia marcescens*, and *Lactobacillus*. No sexually transmitted disease (STI) was found in our study.

## 4. Discussion

To the best of our knowledge, this is the largest study that investigated pediatric AE combining clinical characteristics and comprehensive laboratory results including urinary and hematological data. In our study, age distribution, pathogen spectrum, and the proportion of ABE and ANBE was similar to the data reported in previous studies [11,12,13]. Compared to adult AE, which is most likely associated with sexually transmitted pathogens or enteric bacterial infection [14,15], the etiologies of pediatric AE are usually idiopathic [10,14]. Past studies have confirmed that AE in childhood may be caused by ascending urinary tract infection due to genitourinary anomalies [16,17]. Viral infection, trauma, or postinfectious inflammation also play an important role [1]. However, in clinical practice nowadays, not all patients with AE receive thorough microbiologic diagnostic tests to identify the infectious agents. Thus, the exact etiology and incidence of pediatric AE remain uncertain.

A number of studies have discovered the bimodal distribution of AE cases in pediatric patient groups, with peaks around infancy and prepubertal age [12,18]. The present study with a larger population supported the previous findings. In addition, we further revealed that those with a confirmed bacterial cause also exhibited a similar age distribution. Although the prevalence of AE showed a slight increase in adolescents, particularly in those over 15 years of age, this patient group exhibited distinct characteristics compared to younger individuals, with ANBE being predominant. This observation may be elucidated by findings from prior studies indicating that STIs, such as *Chlamydia trachomatis* and *Neisseria gonorrhoeae*, constitute a substantial proportion of idiopathic or non-bacterial cases in young adults [14,15]. Therefore, further investigation is warranted to ascertain whether additional microbiological assessments for STIs in adolescent AE patients would be beneficial and to determine whether these cases should be treated as pediatric or adult AE.

A recent meta-analysis study that identified 1496 patients with pediatric AE reported that only 10.8% of patients with comprehensive data tested positive for urine culture, although the positive rates varied among different studies [11]. However, 81.7% of these patients received antibiotics. Antibiotic resistance rates worldwide have surged in recent years, particularly with fluoroquinolones and cephalosporins [19,20,21], raising concerns about the overprescription of antibiotics.

There is still no consensus on whether pediatric AE patients should be routinely treated with antibiotics. Traditionally, studies tended to suggest that all boys with AE should be managed as children with urinary tract infection (UTI) to prevent subsequent recurrent infection and renal damage [18,22]. More recent studies, however, suggested more conservative strategies, since only a small proportion of patients had positive urine culture results. As a result, prophylactic antibiotics may be unnecessary in treating idiopathic or single-episode epididymitis in prepubertal boys who have no urological tract anomalies or pyuria. It should only be considered for patients with risk factors that indicate a high likelihood of bacterial infection [10,23].

A study that identified 140 AE cases in pediatric emergency revealed that boys diagnosed with ABE were not significantly different from those with negative urine culture in age, number of WBC on urinalysis or maximum temperature [23]. However, the number of ABE cases in this study was relatively small.

While urine culture remains the gold standard for diagnosing ABE, its lengthy processing time limits its effectiveness for early treatment in clinical settings. Furthermore, as urinalysis relies on indicators including elevated levels of WBCs or leukocyte esterase in various infectious or inflammatory scenarios, it may lead to false-positive results when diagnosing pediatric ABE, since AE in this patient group is often associated with viral infections or idiopathic causes [10,14]. Therefore, identifying and validating key clinical risk factors for predicting positive urine culture results is particularly crucial.

Serum CRP, a highly sensitive acute-phase inflammatory protein, can increase in concentration by up to 1000-fold in response to certain bacterial infections, driven by enhanced genetic expression stimulated by cytokines though various immune pathways [24]. Thus, it has been widely used in clinical conditions as an effective diagnostic tool for evaluating acute inflammation, especially at various bacterial infection sites [25]. Furthermore, serum CRP has been demonstrated to have potential in assisting the diagnosis of infection localization in UTIs, predicting the prognosis of febrile UTIs, and differentiating between inflammatory and non-inflammatory causes of acute scrotum [26,27,28,29,30]. Our study was the first to assess the predictive capability of serum CRP for estimating ABE in pediatric AE. Our analysis further elucidated that CRP was independently associated with ABE and CRP, with a cutoff value of 1 mg/dL showing a high predictive capability for pediatric ABE.

In the present study, a huge discrepancy was discovered between the proportion of patients with ABE and those who received antibiotic therapy. Our findings confirm the robust predictive capability of CRP in differentiating bacterial epididymitis from other etiologies of AE, with the AUC reaching 0.82. From our perspective, given its advantages of high accessibility, time efficiency, and accuracy, serum CRP measurement should be widely adopted as a first-line diagnostic tool in clinical settings to guide the prescription of prophylactic antibiotics and could be incorporated into the future standard of care for pediatric AE.

Our study has several limitations. Firstly, the retrospective design of this study may introduce potential selection biases. Furthermore, inter-individual variability in baseline serum CRP levels may have contributed to a certain level of bias in our results [31]. Secondly, the data regarding laboratory results and clinical manifestations were extracted from medical records, which may result in missing information. Third, the patients in this study were collected from a single center. Further validation analyses involving cohorts from other centers or ethnicity are required. Fourth, we did not compare the predictive capability of different biomarkers (i.e., procalcitonin), which have demonstrated efficacy in evaluating bacterial infections [28,29]. However, CRP has advantages in cost-effectiveness and rapid reaction time, which enable its potential for widespread adoption in routine clinical practice. Further validation studies, threshold analyses, and comparative studies evaluating the prognosis of conservative versus current antibiotic treatment in randomized trials are necessary.

## 5. Conclusions

This study highlighted a substantial discrepancy between the percentage of patients diagnosed with ABE and those who received antibiotic treatment. Our findings demonstrated that CRP was a more accurate predictive tool than urinalysis for distinguishing between ABE and ANBE, providing essential guidance for practitioners when considering antibiotic prescriptions for pediatric AE patients.

## Figures and Tables

**Table 1:** Clinical characteristics, signs, and laboratory results of acute epididymitis.

		All Patients (*n* = 289)	ANBE (*n* = 115)	ABE (*n* = 52)	*p* Value
Age, years		9.5 ± 5.2	9.3 ± 5.5	6.8 ± 5.2	0.02 *
Laterality (*n* = 247)					0.46
	Right	117 (47.4)	49 (48.5)	23 (45.1)	
	Left	114 (46.2)	46 (45.5)	22 (43.1)	
	Bilateral	16 (6.5)	6 (5.9)	6 (11.8)	
Concomitant diseases					0.69
	Urological	57 (19.7)	25 (21.7)	13 (25.0)	
	Non-urological	30 (10.4)	13 (11.3)	8 (15.4)	
Recurrence Symptoms/signs		34 (11.8)	18 (15.7)	10 (19.2)	0.36
	LUT syndrome	5 (1.7)	3 (2.6)	2 (3.8)	0.53
	Scrotal swelling	198 (68.5)	84 (73.0)	41 (78.8)	0.37
	Scrotal erythema	142 (49.1)	67 (58.3)	27 (51.9)	0.09
	Tenderness of epididymis	222 (76.8)	97 (84.3)	43 (82.7)	0.02
Body temperature, °C (*n* = 117)		36.8 (36.5–37.2)	36.8 (36.6–37.2)	36.8 (36.5–37.3)	0.81 *
Fever (>38 °C) (*n* = 117)		17 (14.5)	11 (15.5)	6 (13.0)	0.54
Antibiotic therapy		216 (74.7)	97 (84.3)	45 (86.5)	0.58
Serum WBC count, 10^3^/μL (*n* = 87)		10.56 (8.09–14.65)	10.42 (7.57–14.09)	10.96 (8.33–15.99)	0.23 *
CRP, mg/dL (*n* = 76)		0.80 (0.09–3.54)	0.25 (0.05–1.49)	3.68 (1.56–8.45)	<0.001 *
RBC ≥ 5/HPF on urine analysis (*n* = 211)		32 (17.5)	14 (14.7)	16 (38.1)	0.003
WBC ≥ 5/HPF on urine analysis (*n* = 210)		53 (25.2)	25 (23.1)	21 (41.2)	0.005

Values are presented as mean ± standard deviations, *n* (%), or median (interquartile range). * Mann–Whitney U test. ABE, acute bacterial epididymitis; ANBE, acute non-bacterial epididymitis; CRP, C-reactive protein; HPF, high-power field; LUT, lower urinary tract; RBC, red blood cells; WBC, white blood cells.

**Table 2:** Univariable and multivariable analyses of factors associated with ABE.

	Univariable Analysis		Multivariable Analysis	
	OR (95% CI)	*p* Value	OR (95% CI)	*p* Value
Age	0.89 (0.83–0.94)	0.001	0.88 (0.78–1.01)	0.07
Laterality				
Right	Reference			
Left	1.16 (0.58–2.31)	0.67		
Bilateral	2.52 (0.67–9.54)	0.16		
Recurrence	1.24 (0.55–3.01)	0.43		
Fever (>38 °C)	1.11 (0.38–3.25)	0.57		
WBC ≥ 5/HPF on urinalysis	2.32 (1.14–4.75)	0.02	2.09 (0.47–9.29)	0.33
Serum CRP ≥ 1mg/dL	43.77 (5.52-379.36)	<0.001	61.96 (6.10-629.01)	<0.001

ABE, acute bacterial epididymitis; CI, confidence interval; CRP, C-reactive protein; HPF, high-power field; OR, odds ratio; WBC, white blood cells.

**Table 3:** Comparison of CRP detection and urinalysis in pediatric AE.

Diagnostic Method	Advantages	Disadvantages
CRP	- High sensitivity for detecting bacterial infections and systemic inflammation - Provides rapid results, allowing for early clinical decision-making	- Relatively invasive and costly - Lack of information on the specific pathogen causing the infection - May be elevated in other inflammatory or infectious conditions
Urinalysis	- Non-invasive and widely available	- Limited sensitivity and specificity for diagnosing bacterial AE
		- Lack of capability to differentiate bacterial AE from idiopathic or viral cases
		- Low prevalence of positive urine cultures among pediatric AE patients with positive urinalysis results (10.8% in meta-analysis)

AE, acute epididymitis; CRP, C-reactive protein.

## Data Availability

The data presented in this study are available on request from the corresponding author.

## Supplementary Materials

The following supporting information can be downloaded at: https://www.mdpi.com/article/10.3390/biomedicines12122866/s1, Figure S1: Flow diagram of patients clinically diagnosed as acute epididymitis; Table S1: Pathogen distribution.
