# Supplementary material for: Prediction of Bacterial Etiology in Pediatric Patients with Acute Epididymitis: A Comparison of C-Reactive Protein and Urinalysis in Terms of Diagnostic Accuracy
Source: Biomedicines. 2024 Dec 17;12(12):2866. doi: 10.3390/biomedicines12122866 (PMC11672928; doi:10.3390/biomedicines12122866)
Supplement: Supplementary file 1 [file biomedicines-12-02866-s001.zip › biomedicines-3298332-supplementary.pdf]

Supplemental materials

Figure S1. Flow diagram of patients clinically diagnosed as acute epididymitis

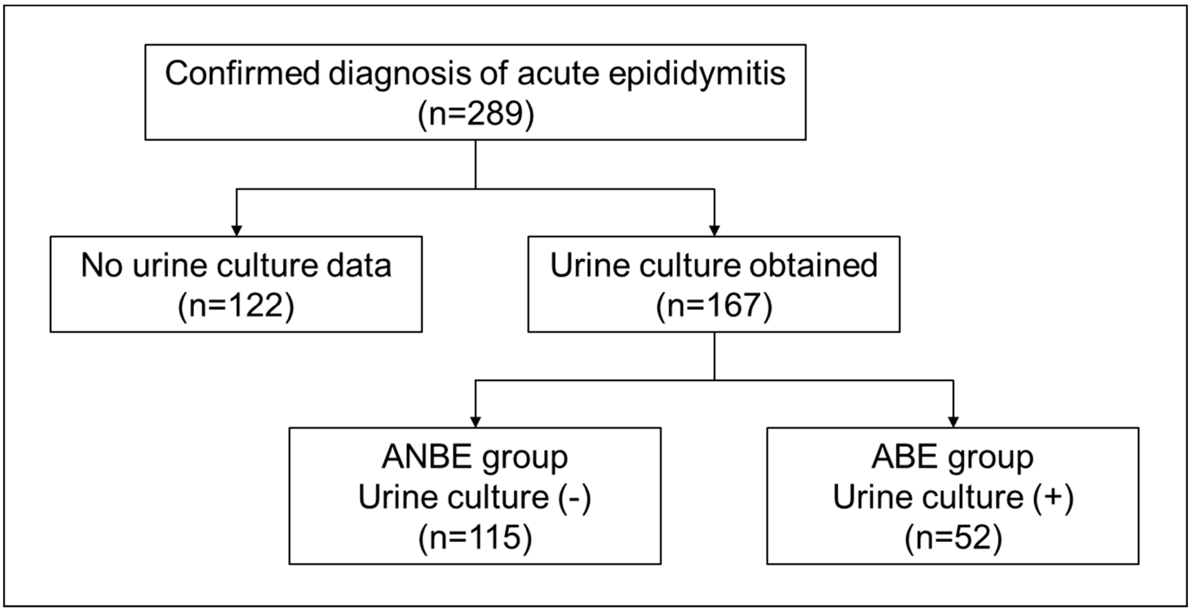

ABE, acute bacterial epididymitis; ANBE, acute non-bacterial epididymitis.

Table S1. Pathogen distribution

Pathogens	No. of cases	Percentage (%)
GNB	31	59.6
Escherichia coli	13	25.0
Pseudomonas aeruginosa	5	9.6
Klebsiella spp.	2	3.8
Citrobacter koseri	1	1.9
Enterobacter cloacae	1	1.9
Morganella morganii	1	1.9
Serratia marcescens	1	1.9
Other	7	13.5
GPC	10	21.7
Enterococcus spp.	4	7.7
Streptococcus viridans	3	5.8
Staphylococcus spp.	2	3.8
Lactobacillus spp.	1	1.9
Multiple pathogens	5	**9.6**
Unknown	6	11.5
**Total**	**52**	**100**

GNB, Gram-negative bacilli; GPC, Gram-positive cocci.
